# Nucleation-controlled growth of superior lead-free perovskite Cs_3_Bi_2_I_9_ single-crystals for high-performance X-ray detection

**DOI:** 10.1038/s41467-020-16034-w

**Published:** 2020-05-08

**Authors:** Yunxia Zhang, Yucheng Liu, Zhuo Xu, Haochen Ye, Zhou Yang, Jiaxue You, Ming Liu, Yihui He, Mercouri G. Kanatzidis, Shengzhong (Frank) Liu

**Affiliations:** 1 Laboratory of Applied Surface and Colloid Chemistry, Ministry of Education; Shaanxi Key Laboratory for Advanced Energy Devices; Shaanxi Engineering Lab for Advanced Energy Technology; Institute for Advanced Energy Materials; School of Materials Science and Engineering, Shaanxi Normal University, Xi’an, 710119 China; 2 Dalian National Laboratory for Clean Energy; iChEM, Dalian Institute of Chemical Physics, Chinese Academy of Sciences, Dalian, 116023 China; 3 University of the Chinese Academy of Sciences, Beijing, 100039 China; 4 Department of Chemistry, Northwestern University, Evanston, IL 60208 United States; 5 Electronic Materials Research Laboratory, Key Laboratory of the Ministry of Education and International Center for Dielectric Research, Xi’an Jiaotong University, Xi’an, 710049 China

**Keywords:** Optical materials, Electronic devices

## Abstract

The organic-inorganic hybrid lead halide perovskites have emerged as a series of star materials for solar cells, lasers and detectors. However, the issues raised by the toxic lead element and marginal stability due to the volatile organic components have severely limited their potential applications. In this work, we develop a nucleation-controlled solution method to grow large size high-quality Cs_3_Bi_2_I_9_ perovskite single crystals (PSCs). Using the technique, we harvest some centimeter-sized single crystals and achieved high device performance. We find that X-ray detectors based on PSCs exhibit high sensitivity of 1652.3 μC Gy_air_^−1^ cm^−2^ and very low detectable dose rate of 130 nGy_air_ s^−1^, both desired in medical diagnostics. In addition, its outstanding thermal stability inspires us to develop a high temperature X-ray detector with stable response at up to 100 °C. Furthermore, PSCs exhibit high X-ray imaging capability thanks to its negligible signal drifting and extremely high stability.

## Abstract (web summary)

Organic-inorganic hybrid halide perovskites have been emerged as promising candidates for X-ray detectors while toxicity and instability are known issues. Here, Zhang et al. grow large size lead-free single crystals for high performance X-ray detection and imaging devices that can operate at 100 °C.

## Introduction

Sensitive X-ray detection is of great significance for broad applications such as therapeutic and diagnostic healthcare, industrial inspection, security screening, scientific research, etc^1,2^. There are two general approaches for X-ray detection, one is indirect conversion using scintillators and the other is direct conversion of X-ray photons into electronic signals. The latter strategy is more advantageous for its higher spatial resolution and simpler system configuration^3^. It is often desired to use reduced X-ray dose rate, particularly for applications related to human and environmental security, therefore it is paramount to develop X-ray detectors with high sensitivity. In this regard, the semiconductor used for direct X-ray detection must exhibit following intrinsic properties: large average atomic number (Z), large carrier mobility-charge carrier lifetime product (*µτ*), and high resistivity (*ρ*). More specifically, the X-ray absorption coefficient, α is determined by Z^4^/*E*^3^ where *E* is the X-ray photon energy^4^. The *µτ* product determines the charge collection efficiency at given electric field^5^. For an X-ray detector, a high signal-to-noise ratio is needed for high sensitivity. Since the shot noise is strongly related to the dark current, an appropriately high resistivity enables a low dark current and, hence, reduces the noise current and increases the signal-to noise ratio, further lowering the detection limit^6^. As for the applications involving human body and biological system, it is apparently desired to reduce radiation dose. Some traditional semiconductors, such as α-Se, have been commercialized for X-ray detection because they can be readily deposited onto thin-film transistor (TFT) to be read out in large flat panels for high space resolution^2,7,8^. However, α-Se is limited by its low *µ*τ (about 10^−7^ cm^2^ V^−1^) and high operating electrical fields (10–40 V μm^−1^), leading to limited sensitivity and detection. Therefore, X-ray detectors with improved sensitivity and lower detection limit are highly desired.

The organic-inorganic hybrid lead-halide perovskites are not only known for their high solar cell efficiency, they are also attractive for radiation detection due to its excellent properties such as high absorption, high *µτ* product and low-cost solution process for fabrication^3,9–12^. A wafer-scale MAPbI_3_ film and MAPbBr_3_ single crystals have been reportedly used in direct X-ray detection with high sensitivities of 2527 µC Gy_air_^−1^ cm^−2^ and 80 µC Gy_air_^−1^ cm^−2^, respectively^13,14^. The integrated MAPbBr_3_-silicon unit also shows an impressive sensitivity of 2.1 × 10^4^ µC Gy_air_^−1^ cm^−2^ under 8 keV radiation^15^. However, with its volatile organic component that associates with instability and toxic lead element that causes environmental concern, its potential is severely limited^16–18^. Among all candidate elements to replace the toxic Pb element, Bi is the most promising for similar electronic structure^19,20^. Hence double-perovskite Cs_2_AgBiBr_6_ single crystals have been grown for X-ray detection^21^, exhibiting detection limit of 59.7 nGy_air_ s^−1^. Unfortunately, its sensitivity is only as low as 105 μC Gy^−1^ cm^−2^, even worse, the largest crystal size obtained using the technique was limited to about three millimeters, too small for more detailed studies, not to mention practical application. More recently, the layered perovskite-like (NH_4_)_3_Bi_2_I_9_ single crystals were developed with high performance of anisotropic response to X-ray^22^, but its long-term stability was limited by its volatile NH_4_^+^. Therefore, it is imperative to develop more suitable materials with low toxicity, suppressed ionic migration, high stability and high sensitivity.

In this work, we develop a nucleation-controlled method to grow centimeter-sized all-inorganic lead-free Cs_3_Bi_2_I_9_ perovskite single crystals (PSCs). The crystals exhibit very low trap state densities, high *μτ* products and low dark current noise. As a result, the Cs_3_Bi_2_I_9_ PSCs X-ray detector shows sensitivity as high as 1652.3 μC Gy_air_^−1^  cm^−2^ and detection limit less than 130 nGy_air_^−1^ s^−1^. Benefitting from the suppressed ion migration, the X-ray detector based on the Cs_3_Bi_2_I_9_ PSC shows exceptional stability in 13 h of continuous operation with superior X-ray imaging capability. In addition, its outstanding thermal stability inspires us to develop a high temperature X-ray detector with stable response at up to 100 °C. It is anticipated that availability of the Cs_3_Bi_2_I_9_ PSCs will inspire research for next generation of X-ray detectors with better performance.

## Results

### Growth of Cs_3_Bi_2_I_9_ PSC

**Fig. 1:**
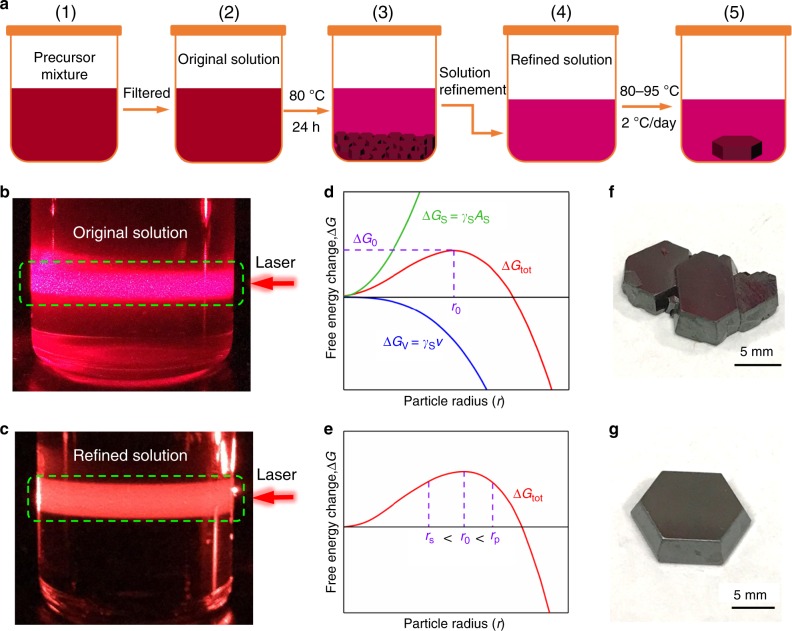
Crystallization of Cs_3_Bi_2_I_9_ perovskite single crystal (PSC). **a** Schematic of the nucleation-controlled method to grow Cs_3_Bi_2_I_9_ PSCs. **b**, **c** The red laser beams illuminate on the original solution and refined solution. **d**, **e** Schematic illustrations of the Gibbs free energy change as a function of particle radius for homogeneous nucleation. (Δ*G*_tot_ is the total Gibbs free energy change due to the change of surface area (Δ*G*s) and the volume (Δ*G*v). Δ*G*_0_ is the free energy change at the critical nucleation point, and *r*_0_ is the critical particle radius). **f**, **g** Photographs of Cs_3_Bi_2_I_9_ PSCs grown using the original solution and refined solution. Scale bar, 5 mm.

The Cs_3_Bi_2_I_9_ adopts a derivative structure of the MX_6_ octahedra, just as in the AMX_3_ perovskite, in which a pair of [BiI_6_]^3−^ octahedra share a common face to form a [Bi_2_I_9_]^3−^ dioctahedral cluster and the voids between the dioctahedral are filled with Cs^+^ cations to forming an isolated zero-dimensional (0D) Cs_3_Bi_2_I_9_ molecular salt structure^23^. It is found that large number of sub-millimeter crystallites can be readily formed shortly after stoichiometric molar ratio of CsI and BiI_3_ powder (CsI:BiI_3_ = 3:2) are mixed in solvent, indicating that the process is nucleation controlled. In order to grow large size single crystals, the number of nuclei has to be critically controlled. In other words, extraneous nucleation seeds, including dusts, bubbles, particulates, defects and scratches on the container surface, etc. need to be removed. For this purpose, we designed a nucleation-controlled method to firstly eliminate the nucleation seeds existed in the system before the intended single crystal growth process. Figure 1a provides an illustration to the process. In brief, 14.04 g CsI and 21.24 g BiI_3_ were dissolved in a 30 mL mixed solvent (DMF/DMSO = 7:3) to prepare precursor with concentration equivalent to 0.6 M Cs_3_Bi_2_I_9_ (Fig. 1a1). After careful filtration, the solution, actually a colloid to be more accurate, was relocated into a temperature-controlled oven (Fig. 1a2. When the system temperature was heated to 80 °C, a large number of sub-millimeter reddish-brown Cs_3_Bi_2_I_9_ crystallites were precipitated on the bottom of the container (Fig. 1a3). To ensure all possible nucleation seeds are removed from the solution, the system was maintained at the temperature for at least 24 hours. At this point, the solution portion is fully saturated and the over-saturated part is recrystallized and precipitated. The upper portion of supernatant is then carefully transferred into another clear container (Fig. 1a4) to grow large single crystals. For the ease of later discussion, this process is referred to as the solution refinement process. Finally, the refined solution was heated from 80 to 95 °C at a ramp rate of 2 °C per day (Fig. 1a5). As a result, a large Cs_3_Bi_2_I_9_ PSC (12 mm × 12 mm × 3 mm) was obtained.

### Nucleation process control

A red laser beam at 635 nm was used to examine the precursor solution to ensure no detectable particulates exist before the crystallization process. As shown in Fig. 1b, the original solution without refinement process shows plenty of particulates under laser illumination. In comparison, after the refinement, the precursor solution shows uniform scattering without observable particulates (Fig. 1c), indicating that nucleation seeds are effectively eliminated. By carefully regulating the temperature from 80 °C to 95 °C, the just-saturated solution became slightly oversaturated to initiate the crystallization on satisfactory nucleation seed. Finally, after about 200 h, large Cs_3_Bi_2_I_9_ PSCs are harvested.

The thermodynamics of the nucleation-controlled method to grow Cs_3_Bi_2_I_9_ PSCs can be explained using Gibbs free energy^5,24^, as discussed in Supplementary Information. As predicted based on the classical nucleation theory, when there are sites with radius *r* larger than the critical nucleation radius *r*_0_ (*r* > *r*_0_), they will grow spontaneously into bigger crystallites or precipitates (as illustrated in Fig. 1d, e). If these crystallites continue to grow, they would reach adjacent ones and merge into a continuous solid, as shown in Fig. 1f. In contrast, after the refinement process removing extraneous nucleation sites, only several large high-quality single crystals with well-defined octahedron are harvested, as shown in Fig. 1g.

**Fig. 2:**
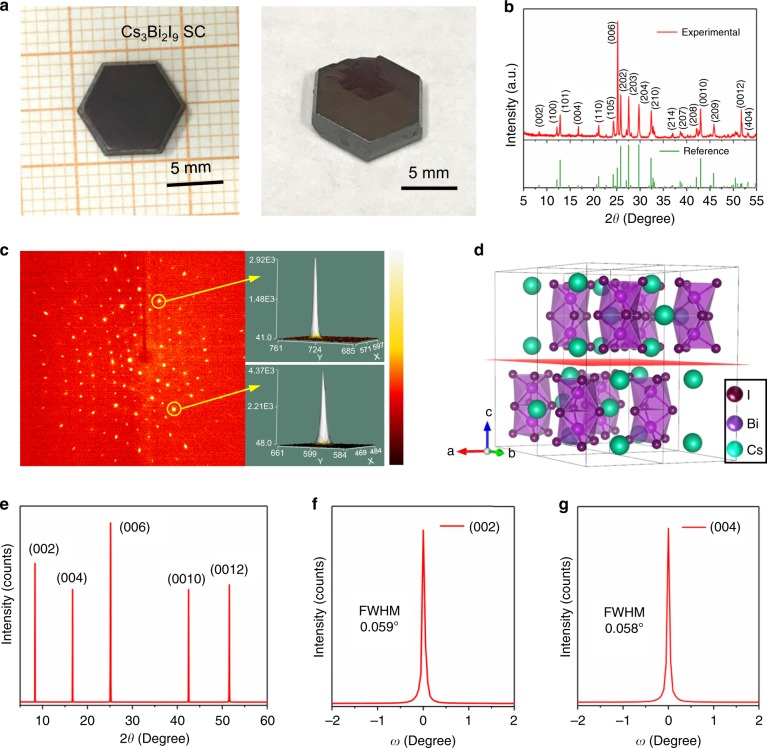
Structural of Cs_3_Bi_2_I_9_ PSC. **a** Photographs of representative Cs_3_Bi_2_I_9_ PSCs. **b** Powder XRD patterns of the Cs_3_Bi_2_I_9_ PSC. **c** Single-crystal X-ray diffraction spots of a Cs_3_Bi_2_I_9_ PSC. **d** Configuration of 2 × 2 × 1 supercell Cs_3_Bi_2_I_9_. **e** XRD 2*θ* scan on the (002) facet of the Cs_3_Bi_2_I_9_ PSC. **f**, **g** High-resolution XRD rocking curves of the diffraction peaks at (002) and (004).

Figure 2a shows the photo taken on well-shaped Cs_3_Bi_2_I_9_ PSCs. The top-view scanning electron microscopy (SEM) image shows only smooth surface with no observable grain boundary (Supplementary Fig. 1a,b), indicating impeccable quality of the single crystal, further demonstrating that the crystal growth method is indeed advantageous. The corresponding SEM-energy-dispersive spectrum (SEM-EDS) analysis shows that the ratio of Cs, Bi and I is 2.58: 2: 7.76 (Supplementary Fig. 1c), consistent with the stoichiometric ratio of Cs_3_Bi_2_I_9_. Additionally, the uniformity of the PSCs is demonstrated by the mapping measurements, as shown in Supplementary Fig. 2). X-ray photoelectron spectroscopy (XPS) was performed to confirm the elemental composition and valence states of the Cs, Bi and I of the Cs_3_Bi_2_I_9_ PSC (Supplementary Fig. 3). According to the survey spectrum of the Cs_3_Bi_2_I_9_ PSC, the elements detected include Cs, Bi, I, O, and C. Note that the O and C originate from carbon dioxide adsorbed from the air during sample preparation. High-resolution XPS spectra for I 3*d*, Bi 4f, and Cs 3d are shown in Supplementary Fig. 3b–d. Peaks for Cs 3*d*_3/2_ (738.4 eV) and 3*d*_5/2_ (724.5 eV), Bi 4*f*_5/2_ (163.9 eV) and 4*f*_7/2_ (158.6 eV), and I 3*d*_3/2_ (630.3 eV) and 3*d*_5/2_ (618.9 eV) can be attributed to the characteristic signals from the Cs^+^, Bi^3+^ and I^–^ species, respectively.

### Structural characterization

X-ray diffraction (XRD) is a powerful tool to probe single-crystalline quality and structural information^25^. Figure 2b shows the powder XRD pattern acquired using a typical Cs_3_Bi_2_I_9_ PSC after being crushed and ground into fine powder. Careful comparison with the library data shows that the crystal adopts a hexagonal structure (space group P6_3_/mmc). This was confirmed by the single crystal diffraction measurement that gives the lattice parameter a = b = 8.39 Å, c = 21.20 Å (the main crystallographic data is presented in Supplementary Table 1). Figure 2c shows the single crystal X-ray diffraction pattern of a Cs_3_Bi_2_I_9_ PSC, in which the well-aligned lattice diffraction spots demonstrate high crystalline quality. Figure 2d presents the hexagonal crystal structure, in which the isolated [Bi_2_I_9_]^3−^ anions (formed by face-sharing of [BiI_6_]^3−^ octahedra anions) filling in the voids formed zero-dimensional (0D) structure. In this hexagonal structure, distinct dimer [Bi_2_I_9_]^3−^ anions placed in a layered arrangement along ab (*00Ɩ*) plane. Therefore, it is considered as a layered crystal structure in the (*00Ɩ*) plane. To further examine the crystalline quality of the Cs_3_Bi_2_I_9_ PSC, high-resolution XRD analysis was carried on a fresh sample. Figure 2e shows five diffraction peaks present at the (002) diffraction plane. A more-detailed rocking-curve analysis shows that the (002) and (004) peaks have very small full width at half maximums (FWHMs), specifically 0.059° and 0.058°, respectively (Fig. 2f,g).

### Optical and transport properties

**Fig. 3:**
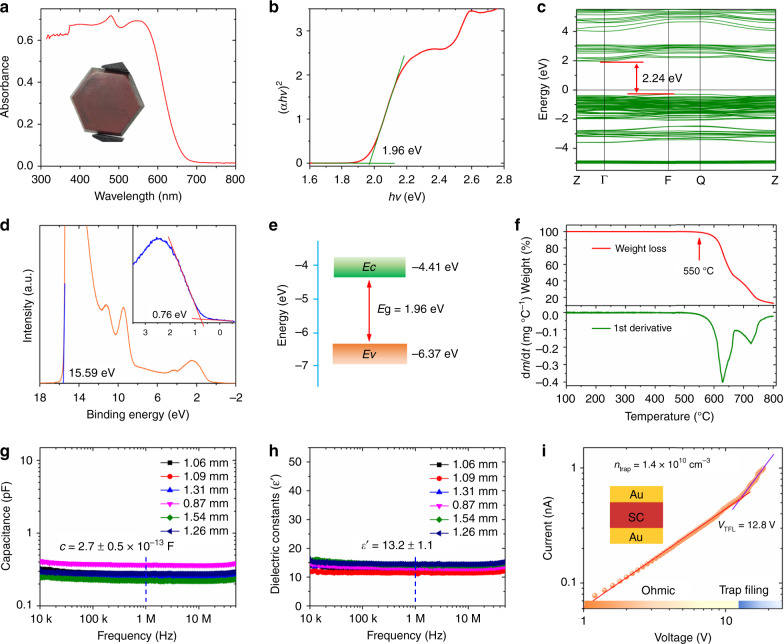
Optical properties and trap state density of Cs_3_Bi_2_I_9_ PSC. **a** Optical absorption spectrum of Cs_3_Bi_2_I_9_. **b** Tauc plot of the Cs_3_Bi_2_I_9_ from the absorption spectrum. **c** Calculated electronic band structure of Cs_3_Bi_2_I_9_ without considering the spin-orbit coupling (SOC) effect. **d** UPS spectrum of the Cs_3_Bi_2_I_9_ PSC. Inset: linear extrapolation in the low-binding-energy region. **e** Energy band diagram of the Cs_3_Bi_2_I_9_ PSC calculated from the Tauc plot and UPS result. **f** Thermogravimetric analysis (mass loss vs. temperature) plot and its first order derivative. **g** Frequency-dependent capacitance and **h** corresponding dielectric constant curves. **i** Current–voltage curve of a hole-only Cs_3_Bi_2_I_9_ PSC device. The top-left inset provides an illustration for the hole-only device structure Au/Cs_3_Bi_2_I_9_ PSC/Au.

To evaluate the potential of the Cs_3_Bi_2_I_9_ PSCs for photoelectronic applications, we measured the optical absorption spectra which show a quite sharp absorption edge at approximately 650 nm, as shown in Fig. 3a. The Tauc plot was calculated from the absorption spectrum (Fig. 3b) displaying a band gap of 1.96 eV, very similar to the value of 1.94 eV reported by Zhang, etc^26^. Figure 3c shows electronic band structure of Cs_3_Bi_2_I_9_ based on the density functional theory (DFT), with a band gap 2.24 eV, in good agreement with prior art results of ~2.30 eV (refs.^23,26^). In addition, the valence and conduction bands are well distributed in energy, which is presented from the projected density of states (Supplementary Fig. 4). The PDOS feature resembles the case of lead-halide perovskites, with the valence band maximum dominated by the Bi 6*s*, I 5*p* states and the conduction band minimum originated from the Bi 6p, I 5p states. Ultraviolet photoelectron spectroscopy (UPS) was also used to determine the Fermi energy (*E*_f_) and the valence band energy (*E*_v_) levels of the Cs_3_Bi_2_I_9_ PSCs, as shown in Fig. 3d. The *E*_f_ was measured to be 5.63 eV, from *E*_f_ = 21.22–15.59, using the cutoff energy (15.59 eV). Using linear extrapolation in the low-binding-energy region, the value of *E*_v_ − *E*_f_ is obtained and the value of *E*v is determined to be 6.37 eV. The conduction band energy (*E*c) is then calculated to be 4.41 eV from (*E*_v_ + *E*_g_). Using above data, the band diagram can be plotted as shown in Fig. 3e (ref. ^27^). Thermal stability was investigated using a thermogravimetric analysis instrument. Figure 3f shows that no detectable mass loss until 550 °C, indicating no decomposition. The thermal stability of the Cs_3_Bi_2_I_9_ PSC is also confirmed by an in-situ XRD measurement, and the results in Supplementary Fig. 5 show that there is no phase change in the XRD pattern. It should be mentioned that the diffraction peaks in the XRD patterns gradually shift toward smaller diffraction angle, because of lattice expansion with temperature increasing gradually from room temperature to 400 °C. Furthermore, the long-term moisture stability of the Cs_3_Bi_2_I_9_ was measured at relative humidity of about 70% without encapsulation. Supplementary Fig. 6 shows that there is no observable change in XRD pattern after being exposed in high humidity for 70 days, demonstrating excellent moisture stability.

In order to identify the trap densities in the Cs_3_Bi_2_I_9_ PSCs, the relative dielectric constants (*ε*) of the Cs_3_Bi_2_I_9_ PSC were estimated from the capacitance-frequency measurement. Capacitance-frequency curves (Fig. 3g) of the Cs_3_Bi_2_I_9_ PSCs were measured using an impedance analyzer, and the relative dielectric constants (Fig. 3h) were calculated from the measured capacitance using the equation $\varepsilon = \frac{{cd}}{{\varepsilon _0A}}$ (where *c* and *ε*_0_ are the capacitance and the vacuum permittivity, and *d* and *A* are the thickness and the electrode area of the Cs_3_Bi_2_I_9_ PSC device, respectively)^6^. After determining the relative dielectric constant of 13.2 ± 1.1, the trap state density of the Cs_3_Bi_2_I_9_ PSC was obtained from the space-charge-limited current (SCLC) method by measuring the dark current–voltage (*I–V*) curve of Cs_3_Bi_2_I_9_ PSC hole-only device. As shown in Fig. 3i, with increasing bias voltage, the current increased from the first linear ohmic region and then a second trap-filled limited (TFL) region. From the second TFL region, the trap density could be calculated using the relation: $n_{{\mathrm{trap}}} = \frac{{2V_{{\mathrm{TFL}}}\varepsilon \varepsilon _0}}{{eL^2}}$ (where *V*_TFL_ is the trap-filled limit voltage, *L* thickness, *ε*_0_ vacuum permittivity, *ε* relative dielectric constant, and *e* electron charge)^28^. The calculated trap density of the Cs_3_Bi_2_I_9_ PSCs is 1.4 × 10^10^ cm^−3^, close to 3D perovskite or 2D perovskite, and significantly lower than the well-known inorganic semiconductors, including polycrystalline Si, CIGS, CdTe, etc^6,29–32^.

**Fig. 4:**
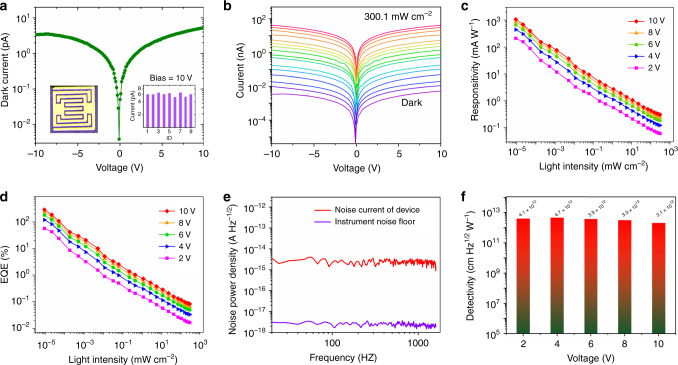
Performance of Cs_3_Bi_2_I_9_ PSC photodetector. **a** Dark *I–V* curve of the Cs_3_Bi_2_I_9_ PSC photodetector with the right-hand inset showing dark current statistics for different devices measured at 10 V bias and the left-hand one device configuration. **b** *I–V* curves of the Cs_3_Bi_2_I_9_ PSC photodetector measured in the dark and with illumination at intensity from 9.2 × 10^−6^ to 300.1 mW cm^−2^, *λ* = 462 nm. **c** Light-intensity-dependent responsivity and **d** external quantum efficiency of the Cs_3_Bi_2_I_9_ PSC photodetector measured at various biases. **e** Measured dark-current noise of the Cs_3_Bi_2_I_9_ PSC photodetector at 4 V bias. **f** Detectivity of the Cs_3_Bi_2_I_9_ PSC photodetector measured at various biases.

The high-quality of the Cs_3_Bi_2_I_9_ PSC along with the good thermal stability (up to 550 °C), superior moisture stability, excellent uniformity and low trap state density point to a highly attractive material for optoelectronic devices. Therefore, planar type photodetectors are designed and fabricated. A pair of interdigitated gold (Au) wires are used as the electrodes, the spacing between adjacent ones is 22 µm and the effective illumination area of each detector is 1.68 × 10^−3^ cm^2^, the device configuration is shown in Fig. 4a.

### Device performance of photodetector

Low dark current is very important for high-performance photodetectors because it helps in achieving high detectivity, responsivity, low noise current and thus large signal-to-noise ratio. Here, we measured the resistivity of the Cs_3_Bi_2_I_9_ SCs. Specifically, a pair of Gold (Au) electrodes (area: 1.8 mm^2^) were deposited using thermal evaporation on the opposite-sides of a crystal with 1.4 mm in thickness. As shown in Supplementary Fig. 7, the calculated resistivity for the Cs_3_Bi_2_I_9_ SC is 2.79 × 10^10^ Ω cm, at least two orders of magnitudes higher than MAPbBr_3_ and other lead-based perovskites (10^7^–10^8^ Ω cm)^13,29^. Note that the high resistivity is desired to reduce background current noise. The dark *I–V* curve of the planar detector was measured, as shown in Fig. 4a. At a 10 V bias, the dark current is as low as 6 pA; such ultra-low dark current is advantageous for detecting very weak signals. Figure 4b shows the *I–V* curves taken in the dark and with illumination at different intensities from 9.2 × 10^−6^ to 300.1 mW cm^−2^. It shows that the photocurrent rises sharply with increasing light intensity (Supplementary Fig. 8), and it reaches 42 nA at 300.1 mW cm^−2^ (462 nm), an increase of 7000 times comparing to the dark current. The responsivity (*R*) is a signature of how effectively the photodetector responds to the optical signal. It is defined by the equation ${{R}} = \frac{{I_{\mathrm{p}} - I_{\mathrm{d}}}}{{A \cdot P}}$, where *I*_P_ is the photocurrent, *I*_d_ dark current, *A* effective illumination area and *P* light intensity^33^. The external quantum efficiency (EQE) represents the photoelectron conversion efficiency defined as $EQE = R\frac{{hc}}{{\lambda e}}$, where *R* represent the responsivity, h the Planck’s constant, *λ* wavelength of incident light, e the elementary charge, and c velocity of light^34^. According to these photocurrent measurements, the corresponding R and EQE were calculated and plotted in Fig. 4c, d. The response is found to increase as the illumination intensity decreases, as more charge recombination is expected under high light intensity. Under the lowest measured incident light intensity, the highest *R* and *EQE* are obtained as high as 1.1 × 10^3^ mA W^−1^ and 288%, respectively, both are among the highest for this type of materials^26,35^.

The detectivity (D*), which characterizes the smallest detectable light signal, is a key figure-of-merit for photodetectors. It is determined by the responsivity and the noise of a photodetector $D^\ast = \frac{{R\sqrt {AB} }}{{i_{\mathrm{n}}}}$, where *R* is responsivity, *A* effective illumination area, *B* electrical bandwidth, and *i*_*n*_ measured noise current. Generally, a low dark current would lead to low noise current^27^. When the noise current is mainly dominated by the shot noise, it may be directly calculated from the dark current. However, in many cases, charge defects in the materials may change the intrinsic noise. It is believed that frequency-dependent noise current is commonly caused by carrier trapping and de-trapping, and it is often higher than the shot noise^36^. Therefore, the exact noise level should be measured from the device rather than calculated simply from dark current. In this work, the noise current of the photodetector was directly measured at different frequencies, as shown in Fig. 4e. It shows that the total noise current measured from the Cs_3_Bi_2_I_9_ PSC photodetector is only 2.2 × 10^−15^ A Hz^−1/2^ at 4 V bias; such a low noise current is expected to result in high detectivity. With the responsivity of the Cs_3_Bi_2_I_9_ PSC photodetector, the peak D* value is calculated to be ~4.7 × 10^12^ cm Hz^1/2^ W^−1^ (Jones), as plotted in Fig. 4f.

### Device performance of X-ray detector

**Fig. 5:**
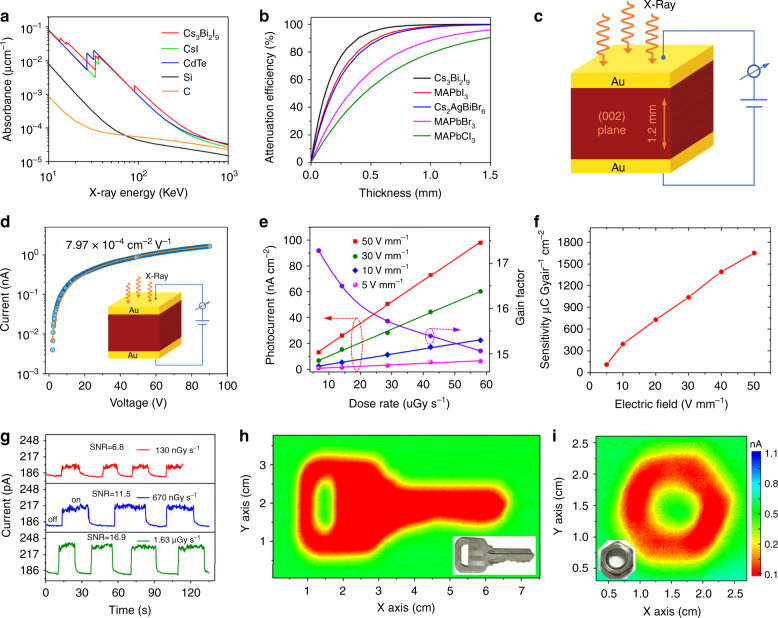
Performance of Cs_3_Bi_2_I_9_ PSC X-ray detector and imaging. **a** Absorption coefficients of Cs_3_Bi_2_I_9_, CsI, CdTe, silicon, and carbon as a function of photon energy. **b** Attenuation efficiency of Cs_3_Bi_2_I_9_, MAPbI_3_, Cs_2_AgBiBr_6_, MAPbBr_3_, MAPbCl_3_, silicon, and carbon for 40 keV X-ray photons versus thickness. **c** Schematic of the Cs_3_Bi_2_I_9_ PSC-based X-ray detector structure. **d** Photoconductivity measurement of the Cs_3_Bi_2_I_9_ PSC device with the inset illustrating the device structure. **e** X-ray-generated photocurrent density and gain factor versus dose rate under different applied biases. **f** Sensitivity under different electric field of the Cs_3_Bi_2_I_9_ PSC X-ray detector. **g** X-ray photocurrent response of the Cs_3_Bi_2_I_9_ PSC device under electric field of 50 V mm^−1^ when exposed to different X-ray dose rates. **h**, **i** Photos and corresponding X-ray images of a key and a nut obtained using the Cs_3_Bi_2_I_9_ SC detector (1 × 1 mm^2^), as measured under 50 V mm^−1^ electric field with dose rate of 36.2 µGy_air_ s^−1^.

Based on the above studies, we conclude that excellent crystal quality and optoelectronic properties has been obtained from the Cs_3_Bi_2_I_9_ PSCs. These excellent properties are also essential for high-performance X-ray detection. For example, the high resistivity, high band gap and ultralow trap density enables an extremely low dark current noise, which is critical for achieving an X-ray detector with a low detection limit. In addition, long-term stability and high temperature resistance indicate this material has great potential for X-ray detection applications. To quantify the X-ray absorption, the absorption coefficient of the Cs_3_Bi_2_I_9_ PSC is obtained as a function of photon energy calculated using the photon cross-section database^37^. As shown in Fig. 5a, it is slightly higher than those of the traditional inorganic semiconductor materials including CsI and CdTe, much higher than those of commercial carbon and silicon materials. Furthermore, the absorption coefficient of the present Cs_3_Bi_2_I_9_ PSC is also higher than those of the popular perovskites studied for X-ray detection^12–15,21,38,39^. The higher the absorption coefficient, the higher the attenuation coefficient. As is evident from Fig. 5b, the all-inorganic lead-free Cs_3_Bi_2_I_9_ PSC has a stronger attenuation than other perovskites at the same thickness. For example, 0.5 mm thick SC is enough for the Cs_3_Bi_2_I_9_ to attenuate 94.7% of the incident X-ray photons, comparing to MAPbI_3_ 87.7%, Cs_2_AgBiBr_6_ 85.7%, MAPbBr_3_ 65.9%, and MAPbCl_3_ 54.2%. Therefore, the high-quality Cs_3_Bi_2_I_9_ PSC was selected to fabricate the vertically structured X-ray detector (Au/Cs_3_Bi_2_I_9_ PSC/Au). As shown in Fig. 5c, the electrode area is 1×1 mm^2^, and the Cs_3_Bi_2_I_9_ PSC thickness is 1.2 mm. The *μτ* product is determined by fitting the modified Hecht equation^27^:

1 $$I = \frac{{I_0\mu \tau V}}{{L^2}}\,\frac{{1 - {\mathrm{exp}}( - \frac{{L^2}}{{\mu \tau V}})}}{{1 + \frac{{Ls}}{{V\mu }}}}$$

where *I*_*0*_ is the saturated photocurrent, and *L* and *V* are the sample thickness and the applied voltage, respectively. In Fig. 5d presents the bias-dependent photoconductivity of the Cs_3_Bi_2_I_9_ PSC. Numerical fitting using the modified Hecht equation gives a *μτ* value of 7.97 × 10^-4^ cm^2^ V^−1^, more than 20 × higher than melt-grown Cs_3_Bi_2_I_9_ crystals^20^ and comparable with other perovskite single crystals^21,27,40^.

A high sensitivity and low detectable dose rate are two vital characteristics required of an X-ray detector for applications. To evaluate the sensitivity, the on/off photocurrent response under various electric field and dose rates are measured, as shown in Supplementary Fig. 9. It is found that the generated photocurrent density shows a linear relationship with the X-ray dose rate, as plotted in Fig. 5e. The slope of a linear fit is defined as the sensitivity of the detector. Figure 5f shows the sensitivity as a function of the electric field applied. At 50 V mm^−1^, the sensitivity is 1652.3 μC Gy_air_^−1^ cm^−2^, more than 3.7 times higher than that of α-Se detectors (440 µC Gy_air_^−1^ cm^−2^) measured under even much higher electric field of 15,000 V mm^−1^ (ref. ^41^). Furthermore, the gain factor G is plotted vs. dose rate used at 50 V mm^−1^ (Fig. 5e, right axis) (the calculation process is provided in Supplementary Information. It should be noted that the gain value gradually increases from 15.1 to 17.3 when the dose rate is decreased from 58.1 to 6.8 µGy_air_ s^−1^. This phenomenon is commonly observed as the dynamic-range enhancing gain compression in photoconductive photodetectors^42^. Furthermore, to find the lowest detectable dose rate, we applied the same method used by Huang et al.^13^. Figure 5g shows the response of the Cs_3_Bi_2_I_9_ PSC X-ray detector collected under different X-ray dose rates at a 50 V mm^−1^ electric field. According to the dark current and photocurrent of a device as shown in Supplementary Fig. 10, the SNR is calculated as 6.8 when the device was exposed under the dose rete of 130 nGy_air_ s^−1^. The international union of pure and applied chemistry (IUPAC) defines the detection limit as the equivalent dose rate to produce a signal greater than 3 times the noise level at a given electric field. Therefore, the lowest detectable X-ray dose rate of the present Cs_3_Bi_2_I_9_ PSC device is 130 nGy_air_ s^−1^, more than two orders of magnitude better than the state-of-the-art MAPbI_3_ SCs (19.1 µGy_air_ s^−1^)^43^ and about 42 times lower than what required for regular medical diagnostics (5.5 µGy_air_ s^−1^)^44,45^. With the ultra-low dark current noise, and highly stable SNR, which guarantee the ignorable baseline drift and highly stable photocurrent output signal, it is expected that the Cs_3_Bi_2_I_9_ PSC device would exhibit excellent imaging capability. As shown in Fig. 5h, i, the images of a metallic key and a nut were reproduced by the X-ray imaging. The distinct color contrast demonstrates that these objects were clearly resolved. For higher resolution imaging applications, the spatial resolution can be further increased by reducing the pixel size and test distance^46^.

### Stability of X-ray detector

**Fig. 6:**
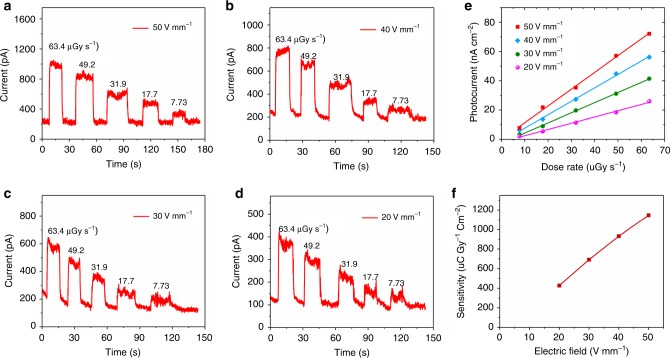
Thermal stability measurement of the Cs_3_Bi_2_I_9_ PSC detector at 100 °C. **a**–**d** ON/OFF photocurrent response under various electric field and dose rates of the Cs_3_Bi_2_I_9_ SC X-ray detector. **e** X-ray generated photocurrent density versus dose rate under different applied electric field. **f** Sensitivity under different electric field of the Cs_3_Bi_2_I_9_ PSC X-ray detector.

Device stability is another important figure-of-merit for practical applications. Supplementary Fig. 11 presents photocurrent response of a Cs_3_Bi_2_I_9_ PSC X-ray detector to the radiation in ambient air under a 30 V mm^−1^ applied electric field. It is clear that when the X-ray is turned on, the photocurrent raises rapidly to the max value 703 pA. As soon as the radiation is off, the current drops to the dark current value 102 pA. Furthermore, there is no detectable photocurrent degradation after being tested in air for the entire period for 50 cycles. In fact, as illustrated in Supplementary Fig. 12, the photocurrent remains unchanged at 724 ± 10 pA for the entire period of 13 h under continuous X-ray illumination and electric field (60 µGy_air_ s^−1^, 30 V mm^−1^). It is also found that the Cs_3_Bi_2_I_9_ PSC device shows long-term stability when exposed to ambient atmosphere. As shown in Supplementary Fig. 13, the response current, resistivity and on/off ratio display no noticeable change after being exposed in ambient for 47 days. Compared with organic-inorganic hybrid perovskite, all inorganic perovskite has better thermal stability. Therefore, we measured the thermal stability of the MA_3_Bi_2_I_9_ SC X-ray detectors under X-ray illumination at different electric field. The on/off photocurrent response under various electric field and dose rates is shown in Fig. 6a–d. Figure 6e presents the generated photocurrent density versus dose rate under different applied electric field when the Cs_3_Bi_2_I_9_ PSC device was maintained at 100 °C. The calculated sensitivity as a function of applied electric field is shown in Fig. 6f. It can be seen that, when at electric field of 50 V mm^−1^, the sensitivity is calculated to be 1146.7 μC Gy_air_^−1^ cm^−2^, which is 69.4% of the value measured at 22 °C, demonstrating that the X-ray detector is capable of being used at high temperature 100 °C. Besides, we find that the detector shows a stable photocurrent response at 100 °C for long time under continuous X-ray illumination and high electric field (63.4 µGy_air_ s^−1^, 50 V mm^−1^), as shown in Supplementary Fig. 14. The surprisingly good stability of the present device can be attributed to its inorganic nature without volatile component, its low ionic migration, and low density of defects^18,47^.

## Discussion

In summary, we have demonstrated a nucleation-controlled method to grow large size high-quality, all-inorganic, lead-free Cs_3_Bi_2_I_9_ perovskite single crystals, which reduces the number of crystal nuclei to ensure that each one grows independently. The Cs_3_Bi_2_I_9_ single crystal shows low trap density, high μτ product, ultra-low dark current noise and high stability. The photocurrent of the Cs_3_Bi_2_I_9_ PSC device was studied under both visible light and X-ray irradiation. The Cs_3_Bi_2_I_9_ PSC photodetector exhibits a high photoresponse on/off ratio (exceeding 10^3^) and high detectivity (10^12^ Jones) to visible light (462 nm). Additionally, high *μτ* product of 7.97 × 10^−4^ cm^2^ V^−1^ was measured, leading to high X-ray sensitivity 1652.3 μC Gy_air_^−1^ cm^−2^, over 3.7 times higher than that of α-Se detectors; and the detection limit of 130 nGy_air_ s^−1^, about 42 times lower than that of required for medical diagnostics. In addition, the Cs_3_Bi_2_I_9_ PSCs exhibit superior X-ray imaging capability thanks to its ultra-low dark current, negligible baseline drift, and extremely high stability. With all above characteristics, we anticipate that the Cs_3_Bi_2_I_9_ may become a promising material in X-ray detection and imaging applications.

## Methods

### Chemicals and regents

N,N-dimethylformamide (DMF, 99.5%) and dimethyl sulfoxide (DMSO, 99.9%) were purchased from Aladdin Reagent Ltd. Bismuth triiodide (BiI_3_, 98%) and cesium iodide (C_S_I, 99%) were purchased from Mackin Biochemical Co., Ltd. All the chemicals were used as received without further purification.

### Crystallization of Cs_3_Bi_2_I_9_ perovskite single crystals (PSCs)

The crystallization procedure is schematically illustrated in Fig. 1a–d. Briefly, 14.04 g CsI and 21.24 g BiI_3_ (3:2 molar ratio) were dissolved in 30 mL DMF/DMSO (7:3) at room temperature under active mixing for 24 h to generate the Cs_3_Bi_2_I_9_ precursor solution (0.6 M). After filtration with an 0.8 μm filter, a brownish red solution was obtained. The filtered precursor solution was heated to 80 °C and the temperature was maintained at least 24 h, until numerous small crystals formed at the bottom of the petri dish. Then, the upper solution from the petri dish was transferred to another petri dish and heated to 95 °C with a slow ramp rate of 2 °C per day. As a result, the desired single crystals were obtained.

### Characterization

X-ray diffraction data were collected using a DX-2700BH with a Cu Kα (*λ* = 1.54186 Å) tube operated at 40 kV and 30 mA. High-resolution X-ray diffraction rocking curve was measured using an X’Pert MRD, with the Cu Kα1 line (λ = 1.5406 Å) with *V* = 40 kV and *I* = 20 mA. The SEM images and EDS were collected with a tungsten filament scanning electron microscope (HITACHI SU-3500) equipped with Energy Dispersive X-ray Spectrometer. The absorption spectra of the Cs_3_Bi_2_I_9_ PSCs were measured using a UV-Vis-NIR spectrophotometer (Lambda 950, Perkin-Elmer). The X-ray Photoelectron Spectroscopy (XPS) and Ultraviolet Photoelectron Spectroscopy (UPS) were measured using a photoelectron spectrometer (ESCALAB 250Xi, Thermo Fisher Scientific). The thermogravimetric analysis (TGA) was implemented on a TA SDT-Q600 V20.9 (Build 20). Capacitances of the Cs_3_Bi_2_I_9_ PSCs were measured using an impedance analyzer (Agilent 4294A Precision LCR Meter) over a wide frequency range from 100 kHz to 30 MHz. The photographs of the Cs_3_Bi_2_I_9_ PSCs were collected using an eight-mega-pixel digital camera.

### Device fabrication and characterization

Several devices were fabricated by depositing Au on two opposite surfaces of the Cs_3_Bi_2_I_9_ PSCs. The vertical Au/Cs_3_Bi_2_I_9_ PSC/Au devices were used to measure the capacitance (Au, 100 nm), trap state density (Au, 100 nm), resistivity (Au, 100 nm) and *μτ* product (Au, 50 nm) along the (002) plane. The *μτ* product was measured under X-ray and the device thickness is 1 mm. Planar-type photodetectors with structure Au/Cs_3_Bi_2_I_9_ PSC/Au were fabricated by depositing interdigitated Au (200 nm) fingers on the Cs_3_Bi_2_I_9_ PSC’s (002) plane. The effective illumination area of each device is approximately 1.68 × 10^−3^ cm^2^, as inserted in Fig. 4a. For the X-ray detectors, 100 nm thick Au coating was deposited on opposite sides of the SCs to form vertical structure Au/Cs_3_Bi_2_I_9_ SC/Au X-ray detectors along (002) plane, the device area (electrode area) is 1 × 1 mm^2^ and 1.2 mm in thickness, as shown in Fig. 5c. The photo-response data of the Cs_3_Bi_2_I_9_ SCs devices were collected using a Keithley 4200 semiconductor characterization system. Dark current noise was measured using a spectrum analyzer (Keysight 35670A). All characterization was conducted in dark with optical and electrical shielding to eliminate the influence from electromagnetic and ambient light.

### X-ray detection performance and imaging measurement

The X-ray detection performance was measured on a home-made measurement system housed in the chamber of an X-ray diffractometer. The tungsten anode X-ray tube (DX-DS2901/24) was used as the source and operated with a constant 40 kV voltage. The operational current was tuned from 40 to 5 mA to adjust the emitted X-ray dose rate. In addition, several pieces of 2-mm-thick Al foils were inserted between the source and the Cs_3_Bi_2_I_9_ SC X-ray detector to serve as the attenuator. The distance between the source and detector is fixed at 16 cm. A metallic optical chopper was used to generate pulsed X-rays. The X-ray dose rate was calibrated using a Fluke Si diode (RaySafe X2 R/F) dosimeter. A Keithley 4200 source meter was used to provide the bias voltage and recorded the response current. All characterization of the X-ray response was conducted in a dark metal chamber to minimize electromagnetic and ambient light disturbance. X-ray imaging capability of the Cs_3_Bi_2_I_9_ SC X-ray detector was measured by moving the objects on a home-built x–y scanning system that can collect the current signal of the detector matched with the object positions. Specifically, the object was fixed on an x–y scanning stage and was allowed to move in and out of the X-ray beam in both the x and y directions to obtain a complete image. A Keysight B2902A source meter connected to the x–y scanning system was used to provide an electric field and record the response current and corresponding position coordinates.

### Computational details

In this work, the density-functional theory (DFT) calculations were performed using the Vienna Ab initio Simulation Package (VASP). The projected augmented wave (PAW) method and the Perdew–Burke–Ernzerhof (PBE) functional within the generalized gradient approximation (GGA) were employed to describe the interaction between ion-cores and valence electrons and the exchange-correlation effects, and an energy cutoff of 500 eV was set for the plane-wave function’s expansion. The van der Waals (vdWs) dispersion correction was applied and described by the DFT-D3 correction. A Γ-centered k-point sampling of 5 × 5 × 2 for Brillouin zone integration was generated using the Monkhorst-Pack scheme during the structural optimization. The lattice parameters and atomic positions of all the structures were relaxed until the total energy changes were less than 1.0 × 10^-5^ eV and the maximum force component acting on each atom was less than 0.01 eV Å^−1^. The electronic properties are calculated based on a denser k-point of 7 × 7 × 3. Normally, due to the self-interaction error inherent in GGA + PBE method, the predication of band gaps of semiconductors is usually much smaller than the accurate values. However, the good agreement between experimental band gaps and theoretical GGA+PBE band gaps has been widely reported for Pb-, Bi-based perovskites. Error cancellation of the underestimation of standard DFT band gap calculation and the overestimation due to the negligence of the spin-orbit coupling (SOC) effects in the heavy element is responsible for this agreement.

### Thermodynamics in the nucleation process

The perovskite nucleation process is rather complex for it is involves different ions^48,49^. At first, is the ions (Cs^+^, Bi^3+^, I^−^) react to form perovskite (Cs_3_Bi_2_I_9_) colloid. Secondly, the colloid particles grow larger to form bulk perovskite crystal. The typical colloid size is around 1.5 nm (ref. ^24^). In colloid science^50^, the colloid particles are often treated as “big atoms” for theoretical consideration. According to the classical nucleation theory, a supersaturated precursor solution will naturally form clusters, tiny crystalline nuclei and precipitates. As illustrated in Fig. 1d, across the critical radius *r*_0_, the change of Δ*G*_total_ goes negative when the particle grows larger. In other words, it becomes a spontaneous process. The *ΔG*_total_, therefore is often referred to as the nucleation barrier. It can be simplified into the sum of the free energy gain due to the new surface generated (Δ*G*s) and the free energy loss (Δ*G*v) associated with the conversion of a unit volume of precursor solution into a solid nucleus^24^:

2 $${\mathrm{\Delta }}G_{total} = 4{\uppi}r^2\gamma + \frac{4}{3}\pi r^3 \cdot \left( { - \varepsilon + \varepsilon _A - k_BT\ln N_A} \right)$$

where *r* is the nucleus radius, *γ* the surface energy per unit area, *ε* the cohesive energy, *K*_B_ the Boltzmann constant, *T* the temperature, and *N*_A_ the mole fraction of isolated molecules. The crystallization of the perovskite will not occur unless a nucleus is provided and it is large enough to overcome the free energy barrier Δ*G*_total_. At the critical point, $d\Delta G_{{\mathrm{total}}}/dr = 0$. Hence, the critical nucleus radius^5^:

3 $${\it{r}}_0 = 2\gamma /(\varepsilon - \varepsilon _{\mathrm{A}} + k_{\mathrm{B}}T\ln \,N_{\mathrm{A}})$$

and the critical free energy barrier:

4 $$\Delta G_{{\mathrm{total}}} = \frac{{16\pi }}{3} \cdot \frac{{\gamma ^3}}{{(\varepsilon - \varepsilon _{\mathrm{A}} + k_{\mathrm{B}}T\ln \,N_{\mathrm{A}})^2}}$$

As shown in Fig. 1d, the maximum of Δ*G*_total_ or Δ*G*_0_ occurs at the critical nucleus radius (*r*_0_). Taking into account the surface free energy gain (Δ*G*s) and the bulk free energy loss (Δ*G*v), the formation of a nucleus in solution is mainly dependent on the critical radius (*r*_0_). In other words, a nucleus with radius (*r*_s_) smaller than the critical radius (*r*_0_) will be dissolved back into the solution, while a larger nucleus with radius (*r*_p_) larger than *r*_0_ are thermodynamically favorable, or it will grow larger spontaneously (Fig. 1e). Thus, the critical nucleus radius is the minimum size at which the particle can grow larger spontaneously in solution.

According to classical nucleation theory, the critical radius of nucleus *r*_0_ is^51^:

5 $$r_0 = 2\gamma /\left| {\Delta \mu } \right|$$

where γ ≈ 40 mN/m is the interfacial energy between solid and liquid of the perovskite^24^ and Δ*μ* the chemical potential difference between solid and liquid of the perovskite. Further, Δ*μ* can be estimated as lattice energy of ionic crystals and typically 1000 kJ m^−3^ (ref. ^51^). Therefore, the estimated perovskite critical radius is around 80 nm.

### Calculation of sensitivity

X-ray sensitivity (*S)* of the detectors can be calculated by the following equation^52^:

6 $$S = \frac{{\Delta I}}{{DA}}$$

where ∆*I* is the photocurrent (∆*I* = *I*_ligh_ *−* *I*_dark_)_,_ *D* the dose rate of incident X-ray radiation, and *A* the area of the detector.

### Calculation of signal-to-noise ratio

The signal-to-noise ratio (*SNR*) was calculated as^13^:

7 $$SNR = \frac{{I_{{\mathrm{signal}}}}}{{I_{{\mathrm{noise}}}}}$$

the signal current (*I*_signal_) is calculated by subtracting the average photocurrent $({\bar I_{photo}})$ by the average dark current $({\bar I_{dark}})$. The noise current (*I*_noise_) is the standard deviation of the photocurrent:

8 $$I_{{\mathrm{noise}}} = \sqrt {\frac{1}{N}\mathop {\sum }\limits_i^N (I_i - \bar I_{{\mathrm{photo}}})^2}$$

### Calculation of gain factor

the gain factor (*G*) of the device was calculated as:

9 $$G = \frac{{I_{\mathrm{R}}}}{{I_{\mathrm{P}}}}$$

where *I*_R_ is the measured current, *I*_P_ is the theoretical current. The theoretical current *I*_P_ is defined as *I*_P_ = *φβe* (ref. ^53^), in which *φ* is the photon absorption rate and *β* the maximum number of carriers generated by a photon. The photon absorption rate *φ* (photons s^−1^) is:

10 $$\varphi = \frac{{\varepsilon Dm_s}}{{E_{{\mathrm{ph}}}}}$$

where *ε* is the fraction of absorbed photons (in this work, *ε* = 100% for the 1.2 mm Cs_3_Bi_2_I_9_ SC), *D* the dose rate, *m*_s_ is the crystal mass, and *E*_ph_ the X-ray energy. The maximum number of photogenerated carrier per photon *β* is calculated as:

11 $$\beta = \frac{{E_{{\mathrm{ph}}}}}{\Delta }$$

where ∆ is the empirical ionization energy, which is calculated by ∆ = 1.43 + 2E_g_ (ref.^54^). Therefore, the theoretical current *I*_P_ can be calculated as:

12 $$I_P = \varphi \beta e = \frac{{\varepsilon Dm_{\mathrm{s}}}}{{E_{{\mathrm{ph}}}}} \times \frac{{E_{{\mathrm{ph}}}}}{{1.43 + 2E_{\mathrm{g}}}} = \frac{{\varepsilon Dm_{\mathrm{s}}e}}{{1.43 + 2E_{\mathrm{g}}}}$$

In this work, ∆ is calculated to be 5.35 eV for Cs_3_Bi_2_I_9_ SC.

### Reporting summary

Further information on experimental design is available in the Nature Research Reporting Summary linked to this paper.

## Supplementary information

Supplementary Information

## Data Availability

The data that support the plots within this paper are available from the corresponding author upon request. The source data underlying Fig. 1b, c, f, g, 2a–g, 3a–d, 3f–i, 4a–f, 5a, b, d–i, 6a–f and Supplementary Figs. 3–14 are provided as a Source Data file.

## Source Data

Source Data
