# Publisher Correction: Nucleation-controlled growth of superior lead-free perovskite Cs_3_Bi_2_I_9_ single-crystals for high-performance X-ray detection

**DOI:** 10.1038/s41467-020-16809-1

**Published:** 2020-06-10

**Authors:** Yunxia Zhang, Yucheng Liu, Zhuo Xu, Haochen Ye, Zhou Yang, Jiaxue You, Ming Liu, Yihui He, Mercouri G. Kanatzidis, Shengzhong (Frank) Liu

**Affiliations:** 1 Laboratory of Applied Surface and Colloid Chemistry, Ministry of Education; Shaanxi Key Laboratory for Advanced Energy Devices; Shaanxi Engineering Lab for Advanced Energy Technology; Institute for Advanced Energy Materials; School of Materials Science and Engineering, Shaanxi Normal University, Xi’an, 710119 China; 2 Dalian National Laboratory for Clean Energy; iChEM, Dalian Institute of Chemical Physics, Chinese Academy of Sciences, Dalian, 116023 China; 3 University of the Chinese Academy of Sciences, Beijing, 100039 China; 4 Department of Chemistry, Northwestern University, Evanston, IL 60208 USA; 5 Electronic Materials Research Laboratory, Key Laboratory of the Ministry of Education and International Center for Dielectric Research, Xi’an Jiaotong University, Xi’an, 710049 China

**Keywords:** Optical materials, Electronic devices

Correction to: *Nature Communications* 10.1038/s41467-020-16034-w, published online 8 May 2020.

The original version of this Article contained the following errors (“Original” column) and the correct terms are shown in the “Corrected” column.

**Table:** 

	Error position	Original	Corrected
1	Fig. 1 title	Fig. 1: Crystallization of Cs3Bi2I9 perovskite single crystal (PSC).	Fig. 1: Crystallization of Cs_3_Bi_2_I_9_ perovskite single crystal (PSC).
2	Fig. 2 title	Fig. 2: Structural of Cs3Bi2I9 PSC.	Fig. 2: Structural of Cs_3_Bi_2_I_9_ PSC.
3	Fig. 3 title	Fig. 3: Optical properties and trap state density of Cs3Bi2I9 PSC	Fig. 3: Optical properties and trap state density of Cs_3_Bi_2_I_9_ PSC
4	Fig. 4 title	Fig. 4: Performance of Cs3Bi2I9 PSC photodetector.	Fig. 4: Performance of Cs_3_Bi_2_I_9_ PSC photodetector.
5	Fig. 5 title	Fig. 5: Performance of Cs3Bi2I9 PSC X-ray detector and imaging.	Fig. 5: Performance of Cs_3_Bi_2_I_9_ PSC X-ray detector and imaging.
6	Fig. 6 title	Fig. 6: Thermal stability measurement of the Cs3Bi2I9 PSC detector at 100 °C	Fig. 6: Thermal stability measurement of the Cs_3_Bi_2_I_9_ PSC detector at 100 °C
7	Fig. 3i	Currenht (nA)	Current (nA)
8	Fig. 4b	300.1 mW cm-2	300.1 mW cm^−2^
9	Fig. 4f	Detectivity (cm Hz^–1/2^w^–1^)	Detectivity (cm Hz^1/2^ W^−1^)
10	Fig. 5f	Sensitivity μC Gy^-2^ cm^-2^	Sensitivity μC Gy^−1^ cm^−2^
11	Fig. 5g	1.63 n Gy s^−1^	1.63 µ Gy s^−1^
12	‘Calculation of signal-to-noise ratio’ in Methods	average photocurrent (퐼̅푝ℎ표푡표) by the average dark current (퐼̅푑푎푟푘).	average photocurrent (*I*_photo_) by the average dark current (*I*_dark_)
13	References 9	CsPbBr3	CsPbBr_3_
14	References 10	SnO2	SnO_2_
15	References 11	CH3NH3PbBr3-xClx	CH_3_NH_3_PbBr_3-x_Cl_x_
16	References 20	A3M2I9	A_3_M_2_I_9_
17	References 21	Cs2AgBiBr6	Cs_2_AgBiBr_6_
18	References 22	(NH_4_)_3_Bi_2_I_9_	(NH_4_)_3_Bi_2_I_9_
19	References 25	C60	C_60_
20	References 26	Cs3Bi2I9	Cs_3_Bi_2_I_9_
21	References 28	CH3NH3PbX3	CH_3_NH_3_PbX_3_
22	References 32	Cu(In,Ga)Se2	Cu(In,Ga)Se_2_
23	References 34	MAPbI3	MAPbI_3_
24	References 35	Cs3Bi2I9	Cs_3_Bi_2_I_9_
25	References 40	Cs2AgBiBr6	Cs_2_AgBiBr_6_
26	References 43	MAPbI3	MAPbI_3_
27	References 52	Cd0.9Zn0.1Te	Cd_0.9_Zn_0.1_Te

This has now been corrected in both the PDF and HTML versions of the Article.

