# Supplementary material for: Nucleation-controlled growth of superior lead-free perovskite Cs3Bi2I9 single-crystals for high-performance X-ray detection
Source: Nat Commun. 2020 May 8;11:2304. doi: 10.1038/s41467-020-16034-w (PMC7210296; doi:10.1038/s41467-020-16034-w)
Supplement: Supplementary file 1 — Supplementary Information [file 41467_2020_16034_MOESM1_ESM.pdf]

Supplementary Information

Nucleation-controlled Growth of Superior Lead-Free Perovskite $Cs_3Bi_2I_9$ Single-Crystals for High-Performance X-ray Detection

Yunxia Zhang[1#], Yucheng Liu[1,4#], Zhuo Xu[1], Haochen Ye[1], Zhou Yang[1], Jiaxue You[1], Ming Liu[3], Yihui He[4], Mercouri G. Kanatzidis[4] and Shengzhong (Frank) Liu[1,2]*

[1]Laboratory of Applied Surface and Colloid Chemistry, Ministry of Education; Shaanxi Key Laboratory for Advanced Energy Devices; Shaanxi Engineering Lab for Advanced Energy Technology; Institute for Advanced Energy Materials; School of Materials Science and Engineering, Shaanxi Normal University, Xi'an 710119, China

[2]Dalian National Laboratory for Clean Energy; iChEM, Dalian Institute of Chemical Physics, Chinese Academy of Sciences, Dalian 116023, Liaoning, China; University of the Chinese Academy of Sciences, Beijing 100039, China

[3]Electronic Materials Research Laboratory, Key Laboratory of the Ministry of Education and International Center for Dielectric Research, Xi'an Jiaotong University, Xi'an 710049, China

[4]Department of Chemistry, Northwestern University, 2145 Sheridan Road, Evanston, Illinois 60208, United States

*Correspondence e-mail: szliu@dicp.ac.cn
#These authors contributed equally: Yunxia Zhang, Yucheng Liu

Supplementary Figures

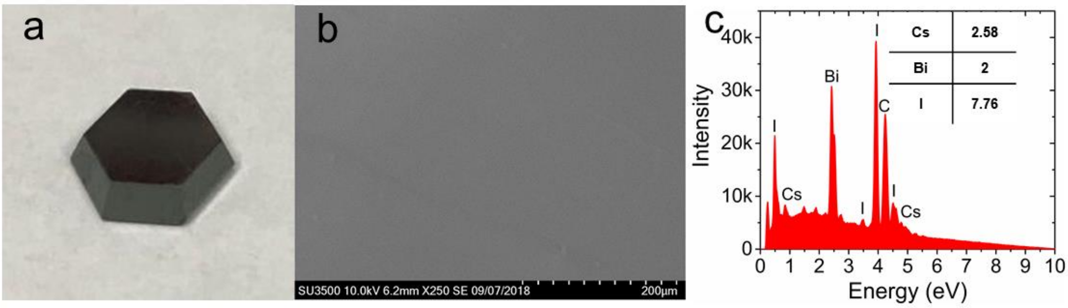

Supplementary Figure 1 (a) Photographs of a $Cs_3Bi_2I_9$ PSC. (b) SEM image of the $Cs_3Bi_2I_9$ PSC surface. (c) EDS spectra of the three elements Cs, Bi and I.

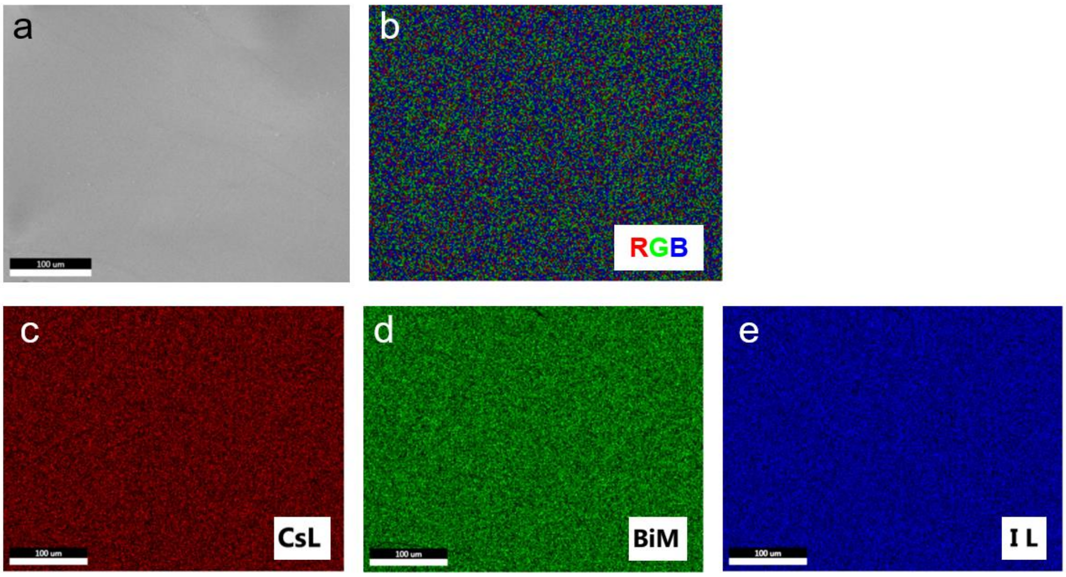

Supplementary Figure 2 EDS mapping scanning measurement of the detected elements Cs, Bi and I.

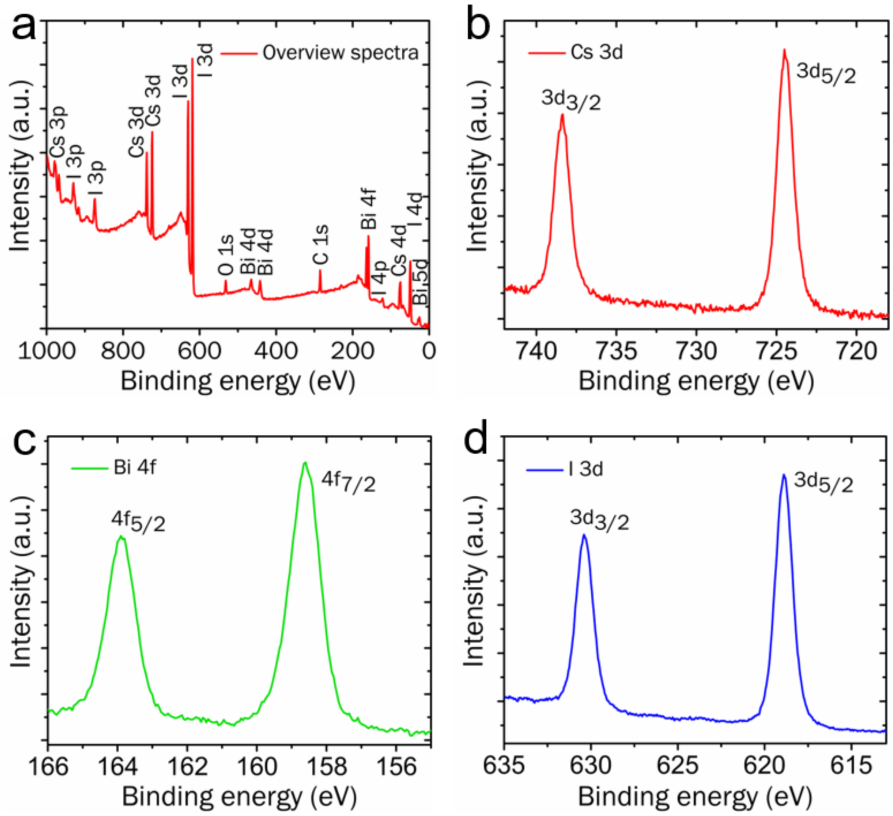

Supplementary Figure 3 (a) XPS overview spectra (b) Cs 3d (c) Bi 4f (d) I 3d regions.

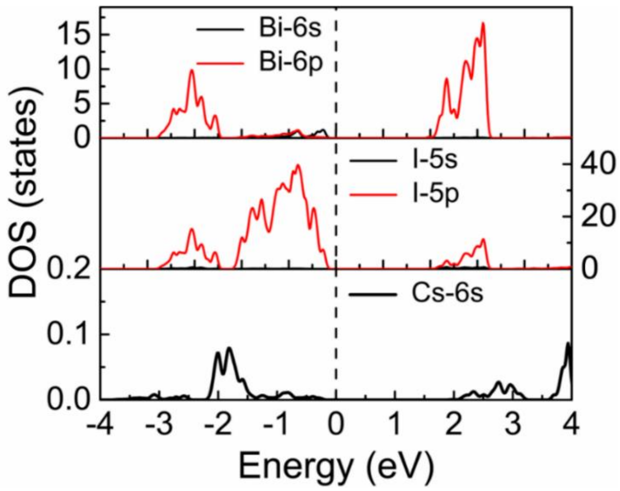

Supplementary Figure 4 The projected density of states of the $Cs_3Bi_2I_9$ s PSCs.

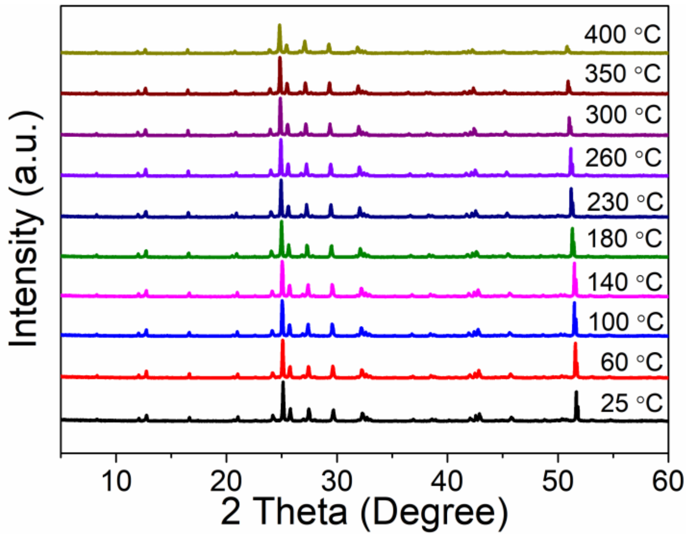

Supplementary Figure 5 The XRD patterns of the $Cs_3Bi_2I_9$ under gradually increasing temperature.

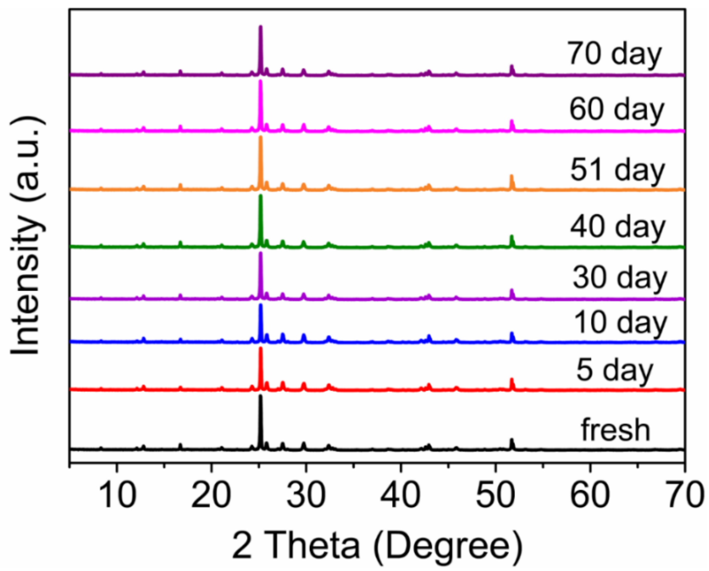

Supplementary Figure 6 The XRD patterns of the $Cs_3Bi_2I_9$, stored in a high humidity environment with ~70 % relative humidity (RH) at 23 °C without encapsulation.

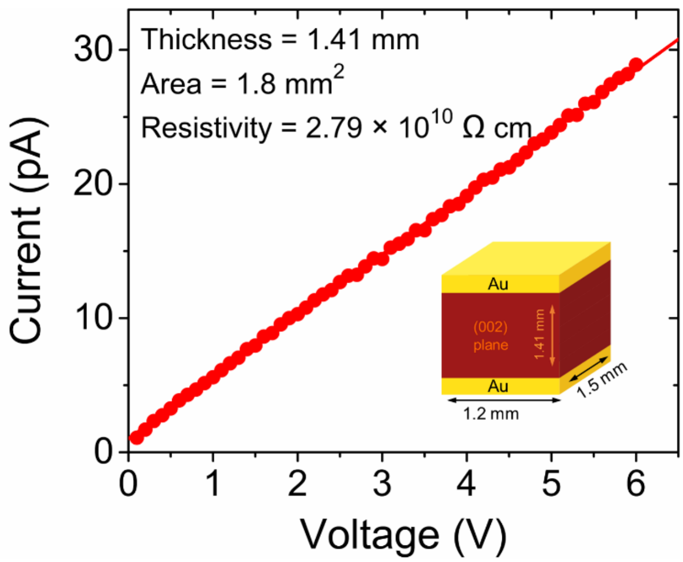

Supplementary Figure 7 Resistivity measurement for $Cs_3Bi_2I_9$ SC, the device configuration and sizes are also inserted.

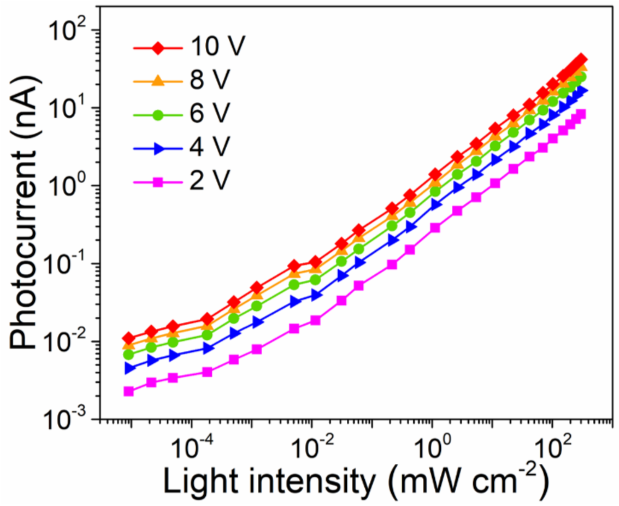

Supplementary Figure 8 Photocurrent of the $Cs_3Bi_2I_9$ PSC device with varying light intensity from 9.2 × 10^{-6} to 300.1 mW cm^{-2} at different biases.

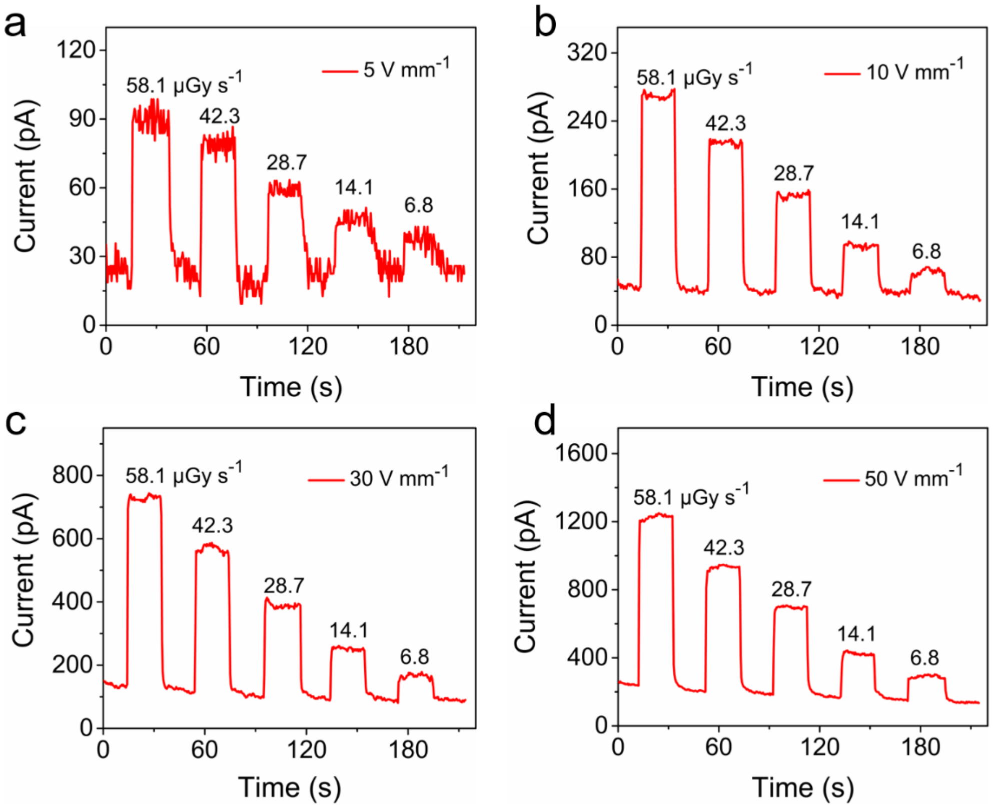

Supplementary Figure 9 ON/OFF photocurrent response under various electric field and dose rates of the $Cs_3Bi_2I_9$ SC X-ray detector.

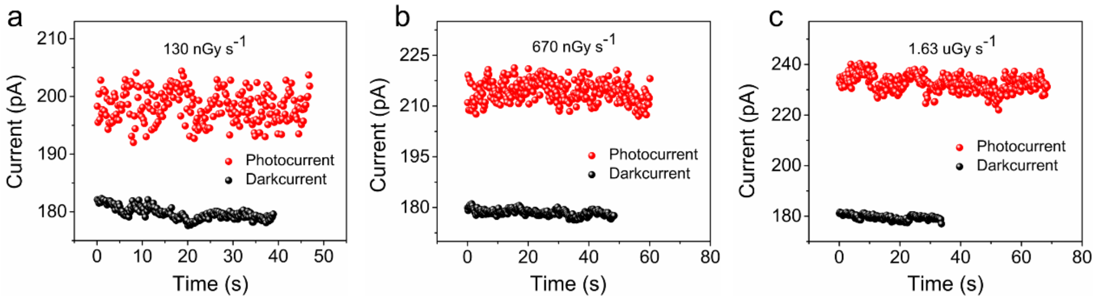

Supplementary Figure 10 Dark current and photocurrent of the $Cs_3Bi_2I_9$ PSC device under different X-ray dose rates at a 50 V mm^{-1} electric field.

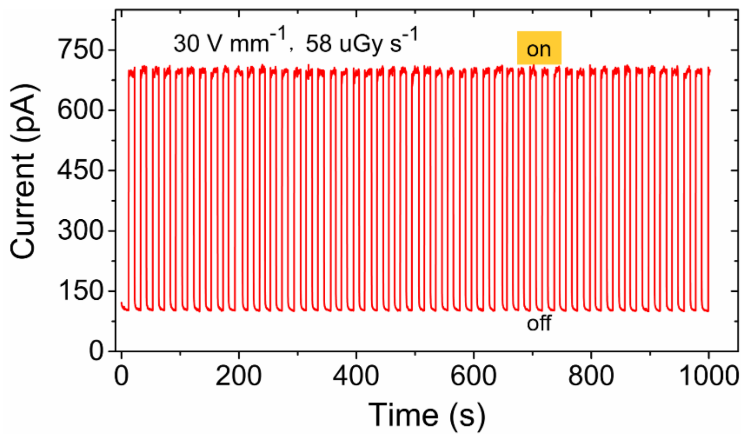

Supplementary Figure 11 $Cs_3Bi_2I_9$ PSC device response to X-rays when turning the X-ray source on and off. The applied electric field was 30 V mm^{-1} and dose rate was 58 uGy s^{-1}.

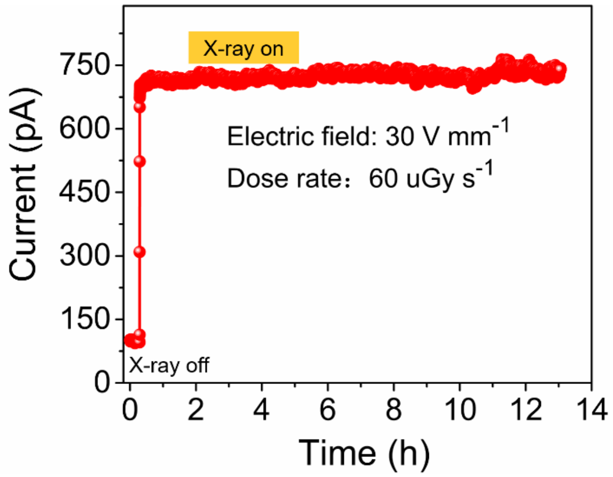

Supplementary Figure 12 Working stability measured for the $Cs_3Bi_2I_9$ PSC detector exposed to X-rays (60 μGy_{air} s^{-1}) at 30 V mm^{-1} electric field.

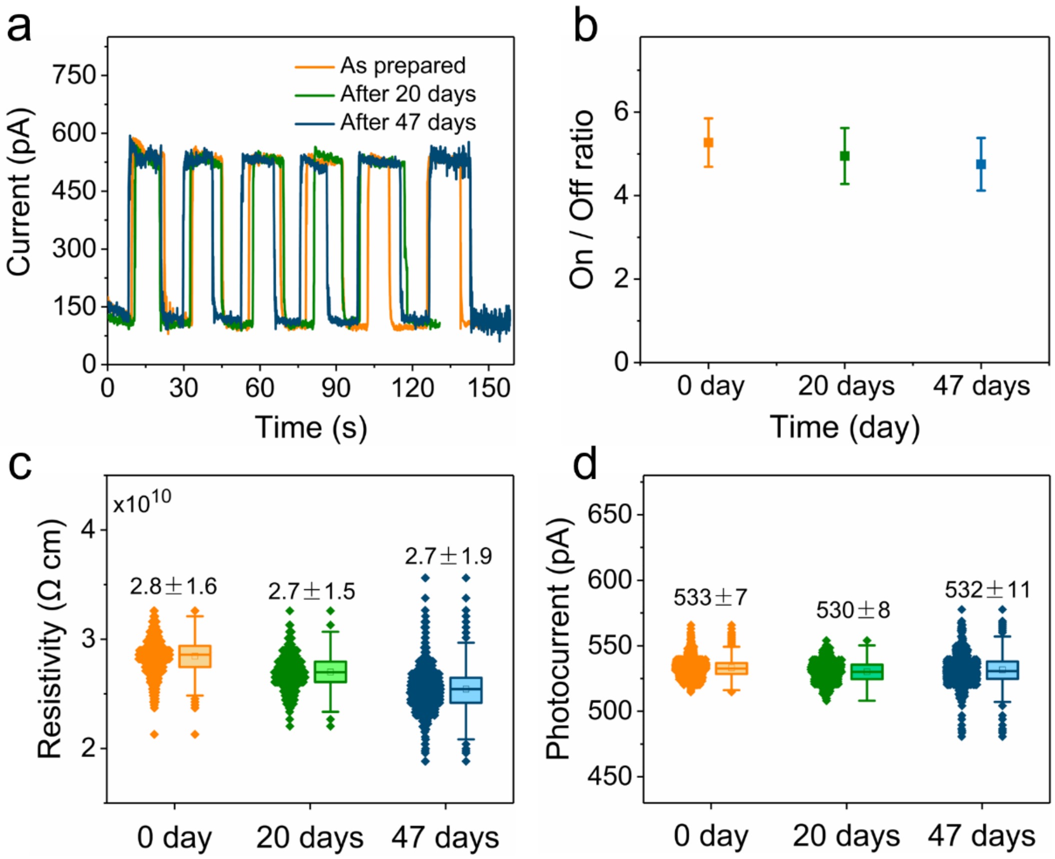

Supplementary Figure 13 Response stability measurement of $Cs_3Bi_2I_9$ SC X-ray detector before and after being exposed in ambient for 47 days. (a) The I-T response curves, (b) On to off ratio, (c) Resistivity and (d) Photocurrent response as a function of exposure time. The response current of the detector was collected at a 30 V mm^{-1} electric field during on-off X-rays illumination switching with a dose rate of 42 μGy_{air} s^{-1}.

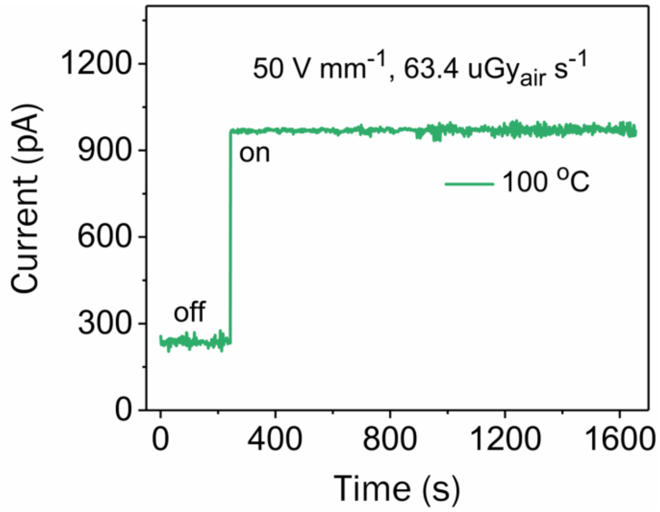

Supplementary Figure 14 Thermal stability measured for the $Cs_3Bi_2I_9$ PSC detector exposed to X-rays (63.4 μGy_{air} s^{-1}) at 50 V mm^{-1} electric field.

Supplementary Table S1. Single-crystal X-ray diffraction data of a $Cs_3Bi_2I_9$ single crystal.

Empirical formula	$Cs_3Bi_2I_9$
Formula weight	1958.8
Temperature	298 K
Wavelength	0.71073
Crystal system	Hexagonal
Space group	$P6_3/mmc$
Unit cell dimensions	a = 8.3903 α = 90°
	b = 8.3903 β = 90°
	c = 21.2023 γ = 120°
volume	1292.60
2θ range for data collection	5.598-53.007°
Reflections collected	10427
Absorption correction	0.2
Max and min transmission	0.7454 and 0.2150
Refinement method	Full-matrix least-squares on F^2
